# Effects of Prey Macronutrient Content on Body Composition and Nutrient Intake in a Web-Building Spider

**DOI:** 10.1371/journal.pone.0099165

**Published:** 2014-06-09

**Authors:** Jesse Hawley, Stephen J. Simpson, Shawn M. Wilder

**Affiliations:** School of Biological Sciences and the Charles Perkins Centre, The University of Sydney, Sydney, NSW, Australia; Aarhus University, Denmark

## Abstract

The nutritional composition of diets can vary widely in nature and have large effects on the growth, reproduction and survival of animals. Many animals, especially herbivores, will tightly regulate the nutritional composition of their body, which has been referred to as nutritional homeostasis. We tested how experimental manipulation of the lipid and protein content of live prey affected the nutrient reserves and subsequent diet regulation of web-building spiders, *Argiope keyserlingi*. Live locusts were injected with experimental solutions containing specific amounts of lipid and protein and then fed to spiders. The nutrient composition of the spiders' bodies was directly related to the nutrient composition of the prey on which they fed. We then conducted an experiment where spiders were fed either high lipid or high protein prey and subsequently provided with two large unmanipulated locusts. Prior diet did not affect the amount or ratio of lipid and protein ingested by spiders when feeding on unmanipulated prey. *Argiope keyserlingi* were flexible in the storage of lipid and protein in their bodies and did not bias their extraction of nutrients from prey to compensate for previously biased diets. Some carnivores, especially those that experience frequent food limitation, may be less likely to strictly regulate their body composition than herbivores because food limitation may encourage opportunistic ingestion and assimilation of nutrients.

## Introduction

The balance of nutrients consumed by animals can have large effects on growth, survival and reproduction [1], [2]. Yet, in nature, the balance of nutrients in food items often does not match the balance of nutrients required by animals to maximize fitness [1], [2]. As a consequence, many animals have evolved mechanisms to regulate their diet and to ensure some degree of nutritional homeostasis, which is the regulation of fairly constant ratios of nutrients in their bodies [3]–[7]. Nutritional homeostasis, and diet balancing in general, can be important mechanisms that allow animals to satisfy particular physiological requirements for nutrients in nutritionally variable environments [1], [6].

Animals can use a variety of behavioral and physiological mechanisms both pre- and post-ingestively to regulate their nutrient status. Pre-ingestively, animals can select among food items or select among different parts of individual food items to regulate the balance of nutrients that they ingest [2]. This has been demonstrated among herbivores that feed on different plants or plant parts and also in carnivores that feed on different parts of animals or selectively consume particular nutrients from animal bodies [4], [5], [8], [9]. Animals can also regulate nutrient storage post-ingestively by selectively assimilating, egesting or metabolizing nutrients [2], [10]. Pre- and post-ingestive diet balancing mechanisms could provide animals with a wide range of opportunities for moderating differences between the balance of nutrients in food and that required for optimal growth, reproduction or survival.

Despite a large number of datasets on the regulation of nutrient reserves in animals [reviewed in 6], there are still significant gaps in our understanding of differences in the degree of nutritional homeostasis among groups of animals. For example, it is unclear if the degree to which animals regulate the balance of nutrients in their bodies varies among trophic levels as the majority of studies have been conducted on herbivores or omnivores. Nutritional homeostasis is an important simplifying assumption for some food web models and it is important to understand if this pattern is observed among both herbivores and carnivores [7].

We tested the effects of prey protein and lipid content on the body composition and subsequent diet regulation of the web-building spider, *Argiope keyserlingi*. In the first experiment, we quantified the nutrient content of spiders that were fed locusts injected with one of three solutions differing in lipid and protein content. Nutritional homeostasis predicts that animals should regulate a constant body composition the value of which may depend on the phase of the life cycle [1], [6]. However, frequent food limitation of spiders may have relaxed selection for the regulation of homeostatic body composition in favor of maximizing nutrient storage [11]. In the second experiment, we fed two lipid- or protein-injected locusts to spiders and then fed them two additional unmanipulated locusts to test if they could bias their extraction of nutrients to remediate prior nutrient imbalances. Several studies have suggested that some spiders can regulate the balance of nutrients that they ingest from prey depending on their prior diet [8], [12]. Yet, the generality of this phenomenon remains unknown.

## Materials and Methods

### Study Species

Experiments were conducted on the St. Andrew's Cross spider, *Argiope keyserlingi*. We collected penultimate female spiders from *Lomandra longifolia* plants on the campus of the University of Sydney (Sydney, NSW, Australia) in October and November of 2010 and 2011. Individuals were fed two locusts (fourth instar) per week until they molted to adulthood. After molting, we weighed females and randomly assigned them to the experimental treatments.

Upon molting to adulthood, female spiders allocate much of their assimilated nutrients to eggs. Eggs could differ slightly in their composition relative to the female body. Hence, as females transition from no eggs to a full ovary, their homeostatic target could change. Whether animals are in steady-state or transition, if animals are regulating body composition through homeostasis, then their body composition should be similar at similar points in time regardless of differences in diet.

### Experimental Manipulation of Prey Nutrients

Both experiments used locusts as prey. The nutrient content of orthopterans falls well within the range observed for other arthropods and orthopterans can be common, if not dominant, prey in the diet of some *Argiope* spp. spiders [13], [14]. We experimentally manipulated the nutrient content of the prey by injecting them with solutions (see below) that varied in the relative amounts of protein (albumin powder mixed in water) and lipid (canola oil). Prior to injecting locusts with the experimental solutions, we removed the hind jumping legs and starved locusts for three days. This reduced variability in nutrient consumption that could occur due to hindlimb autotomy during prey capture (i.e., loss of a protein-rich leg). Locusts were either treated with a sham injection or injected with 0.06 mL of solution immediately prior to being fed to spiders. For the injection, the needle was inserted in the posterior end of the abdomen, the injection was made, and, immediately after removing the needle, thread was tied around the injection site (i.e., 1–3 mm from the posterior end of the abdomen) on each locust to prevent haemolymph and solutions from leaking.

### Prey Nutrients and Spider Body Composition

In this experiment, fourth instar locusts, *Locusta migratoria*, (ca. 200–300 mg) were used as prey. Locusts were reared in gregarious cultures and fed with wheatgrass and dry wheat germ. Three treatment solutions were prepared: Low Protein (2.5 g albumin in 15 mL of water), High Protein (7.5 g of albumin in 15 mL of water), and High Lipid, Low Protein (5 mL canola oil and 2.5 g albumin in 15 mL of water). The small amount of protein was added to the High Lipid treatment to help solubilize the oil in the water. A fourth treatment of a sham injection was also included in which locusts were pierced by a needle but nothing was injected. Each locust was injected with 0.06 mL of solution or the sham treatment and placed in the spider's web while still alive.

For each treatment, we fed spiders one locust on two occasions spaced three days apart and then euthanized spiders five days after their second feeding. This five-day period should have been sufficient to allow complete digestion and assimilation of the last meal but avoid starvation and use of nutrient reserves [15], [16].

We measured the lipid and protein content in the bodies of spiders and control locusts (i.e., injected but not fed to spiders) for each treatment. Lipid content was measured gravimetrically using chloroform as a solvent and protein content was measured using the Bradford assay modified for use in 96-well microplates (Bio-Rad Protein Assay Kit, Bio-Rad, Australia). While these assays were not able to distinguish the source of the nutrients (e.g., nutrient reserves, eggs, etc.), they still allowed us to test the predictions of nutritional homeostasis, which is based on whole-body nutrient composition [1], [6].

We first used multivariate analysis of variance with both lipid and protein content as response variables to test the effects of the diet treatments on the body composition of locusts and spiders. The concentrations of protein and lipid injected into locusts were used as independent variables. We then conducted separate one-way analysis of variance to identify the nature of the multivariate effects (i.e., separate analyses of lipid and protein for locusts and spiders). We also used linear regression to compare the lipid:protein composition of locusts with that of spiders.

### Prey Nutrients and Diet Regulation

Locusts were used for four rounds of feeding: two feedings with experimentally injected locusts (Australian plague locusts, *Chortoicetes terminifera*, ca. 120–220 mg) followed by two feedings with unmanipulated locusts (*Locusta migratoria*). Feedings were spaced four days apart. Fourth-instar *Chortoicetes terminifera* were reared under the same conditions as the *L. migratoria* used in the previous component. Two experimental solutions were prepared: High Protein (10 g albumin and 2 mL canola oil in 15 mL water) and High Lipid (2 g albumin and 10 mL canola oil in 15 mL water). We changed the solutions for this experiment to ensure that both treatments were given both albumin and canola oil but in different ratios. We injected each locust with 0.06 mL of solution using the same procedure as the previous experiment. Fourth-instar *Locusta migratoria* were used as the unmanipulated locust prey and were taken directly from the cultures on the morning of the feeding with all legs were left intact. Locusts used for the injection feedings (165+10 mg) were smaller than those used during the unmanipulated feedings (366+11 mg) to ensure that spiders were not satiated from feeding on the injected prey and to provide an unmanipulated prey that was large enough to allow spiders to selectively extract nutrients from the prey body. We removed the remains of locusts after spiders had finished feeding to quantify the nutrients spiders had extracted from each locust [17], [18]. Nutrients were quantified using the same assays as in the first experiment.

To test if nutrients were differentially extracted from the bodies of unmanipulated locusts, we compared the consumption of lipid and protein by spiders in the two diet treatments using analysis of covariance. In these analyses, prior diet treatment was an independent variable, the amount of each nutrient present in the unmanipulated locust's bodies was a covariate, and the amount of each nutrient consumed by spiders was the response variable. If spiders were differentially extracting nutrients, then we would predict that the relationship between nutrients present and nutrients consumed (i.e., the proportion of available nutrients consumed) by spiders would differ between high lipid and high protein treatments. Separate analyses were conducted for the first and second unmanipulated locusts and separately for lipid, protein, and the ratio of lipid to protein consumed by spiders.

## Results

### Prey Nutrients and Spider Body Composition

**Figure 1:**
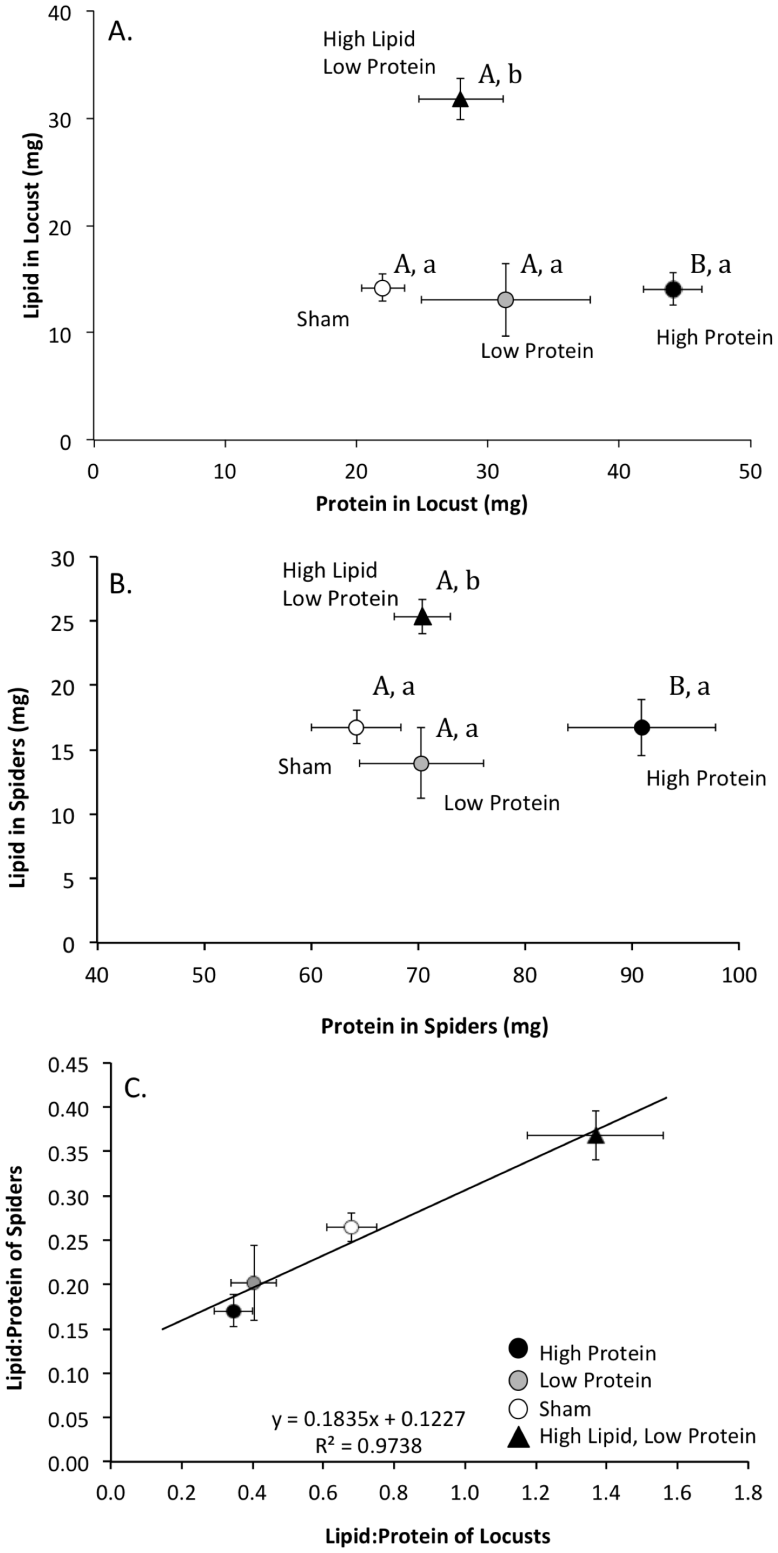
Nutrients in the bodies of locusts and spiders. Comparisons of the effects of macronutrient injection treatments on A) the lipid and protein content of locusts, *Locusta migratoria*, B) the lipid and protein content of spiders, *Argiope keyserlingi*, that fed on the locusts, and C) the relationship between lipid:protein in locusts and spiders that fed on them. In A and B, treatments with different letters are significantly different from each other in protein (capital letters) and lipid (lower case letters). Values are the mean +1 SE.

The nutrient injection treatments changed the nutrient content of locust bodies as expected (MANOVA: effects of lipid injection: Wilks' lambda = 0.52, F_2,44_ = 20.4, p<0.001; effects of protein injection: Wilks' lambda = 0.54, F_2,88_ = 7.9, p<0.001). Injections of protein into locusts resulted in significant increases in their protein content (F_2,48_ = 18.6, p<0.001) with no change in lipid content (F_2,48_ = 0.08, p = 0.93; Figure 1A) relative to sham controls. Injections of lipid and small amounts of protein resulted in significant increases in the lipid content (F_1,48_ = 39.5, p<0.001) of locusts but no significant increase in their protein content (F_1,48_ = 0.5, p = 0.48; Figure 1A) relative to sham controls.

The lipid and protein content of the spider's bodies was significantly affected by the nutrients injected into their locust prey (Figure 1B; MANOVA: effects of lipid injection: Wilks' lambda = 0.52, F_2,44_ = 20.4, p<0.001; effects of protein injection: Wilks' lambda = 0.54, F_2,88_ = 7.9, p<0.001). Spiders fed protein-injected locusts had higher protein (F_2,46_ = 7.9, p<0.001) and no difference in lipid content (F_1,46_ = 0.4, p = 0.64) in their bodies compared to sham-fed spiders. Spiders fed lipid-injected locusts had higher lipid (F_1,46_ = 13.8, p<0.001) and no difference in protein content (F_2,46_ = 0.0, p = 0.99) in their bodies compared to sham-fed spiders. Patterns in spider body composition reflected the same patterns observed in the composition of locusts on which they fed (Figure 1A, B). There was also a very strong linear relationship (r^2^ = 0.97) between the lipid:protein in the bodies of locusts and the lipid:protein in the bodies of spiders that fed on those locusts (F_1,3_ = 74.3, p = 0.01; Figure 1C).

### Prey Nutrients and Diet Regulation

**Figure 2:**
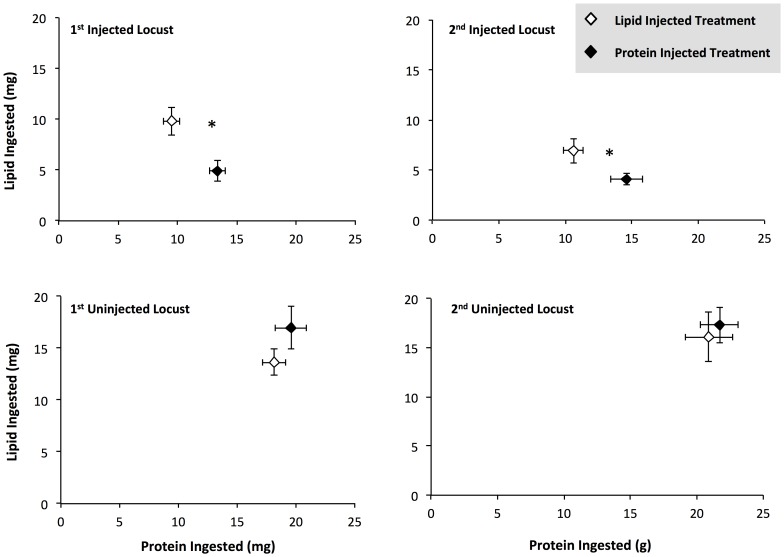
Nutrient consumption by spiders in the differential extraction experiment. Comparisons of the consumption of lipid and protein by *Argiope keyserlingi* spiders when feeding on sequential locusts, *Chortoicetes terminifera*: A) the first locust injected with nutrient solutions, B) the second locust injected with nutrient solutions, C) the first unmanipulated locust, and D) the second unmanipulated locust. Values are the mean +1 SE. * denote significant multivariate and univariate differences in protein and lipid consumption between treatments.

For the first two feedings, when spiders were fed the experimentally manipulated locusts, spiders on the high lipid treatment consumed significantly more lipid and less protein that spiders on the high protein treatment (Figure 2A,B; Table 1; MANOVA: 1^st^ injected locust: Wilks' Lambda = 20.6, F_2,25_ = 20.6, p<0.001; 2^nd^ injected locust: Wilks' Lambda = 9.8, F_2,24_ = 9.8, p = 0.001). However, when spiders were fed the first (Wilks' Lambda = 0.9, F_2,25_ = 1.3, p = 0.28) and second (F_2,20_ = 0.007, p = 0.99) unmanipulated locusts, there were no significant differences in lipid and protein consumption between spiders on the two treatments (Figure 2C, D; Table 1).

**Table 1:** Statistical results for nutrient extraction experiment.

			Effect of Injection Treatment		
Locust Number	Nutrient Eaten		df	F	p
1st Injected Locust	Protein		1,26	16.8	**<0.001**
	Lipid		1,26	7.6	**0.01**
2nd Injected Locust	Protein		1,25	7.7	**0.01**
	Lipid		1,25	5.8	**0.02**
1st Unmanipulated Locust	Protein		1,26	0.8	0.36
	Lipid		1,26	2.0	0.16
2nd Unmanipulated Locust	Protein		1,22	0.1	0.95
	Lipid		1,22	0.1	0.91

The results of univariate analysis of variance testing the effects of injection treatment (High Protein or High Lipid) on the mass of protein or lipid consumed by spiders when feeding on locusts.

**Figure 3:**
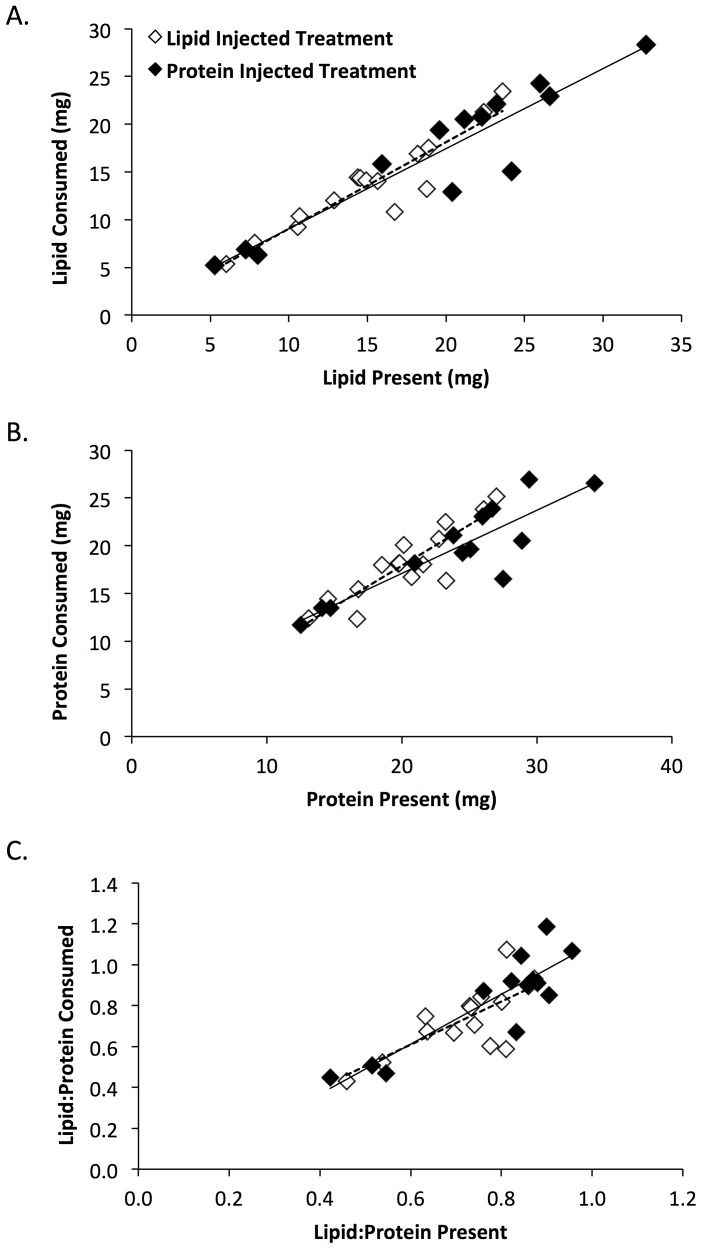
Nutrient extraction by spiders feeding on unmanipulated locust 1. For the first unmanipulated locust, tests for differential extraction of A) lipid, B) protein, and C) the ratio of lipid to protein from the locust body by *Argiope keyserlingi* spiders. All spiders had previously been fed two locusts that were injected with nutrient solutions. Regression lines were plotted separately for spiders previously fed lipid injected locusts (dashed line) and spiders previously fed protein injected locusts (solid line).

There was no evidence for differential consumption of lipid or protein by spiders on the two diet treatments. For the first unmanipulated locust, there were no differences between diet treatments in the relationships between lipid present in a locust and lipid consumed by the spider (F_1,24_ = 0.3, p = 0.61), protein present in the locust and protein consumed by the spider (F_1,24_ = 1.9, p = 0.18) and the ratio of lipid to protein present in a locust and that consumed by the spider (F_1,24_ = 0.4, p = 0.55; Figure 3). For the first unmanipulated locust, the relationships between nutrients present in the locust body and nutrients consumed by spiders were linear (Figure 3; protein: F_1,24_ = 77.8, p<0.001; lipid: F_1,24_ = 142.1, p<0.001; ratio: F_1,24_ = 45.6, p<0.001). Spiders consumed a high and fairly constant proportion of available nutrients from these locusts (Figure 3; mean +1 SE; 87+2% of available protein, and 90+2% of available lipid).

**Figure 4:**
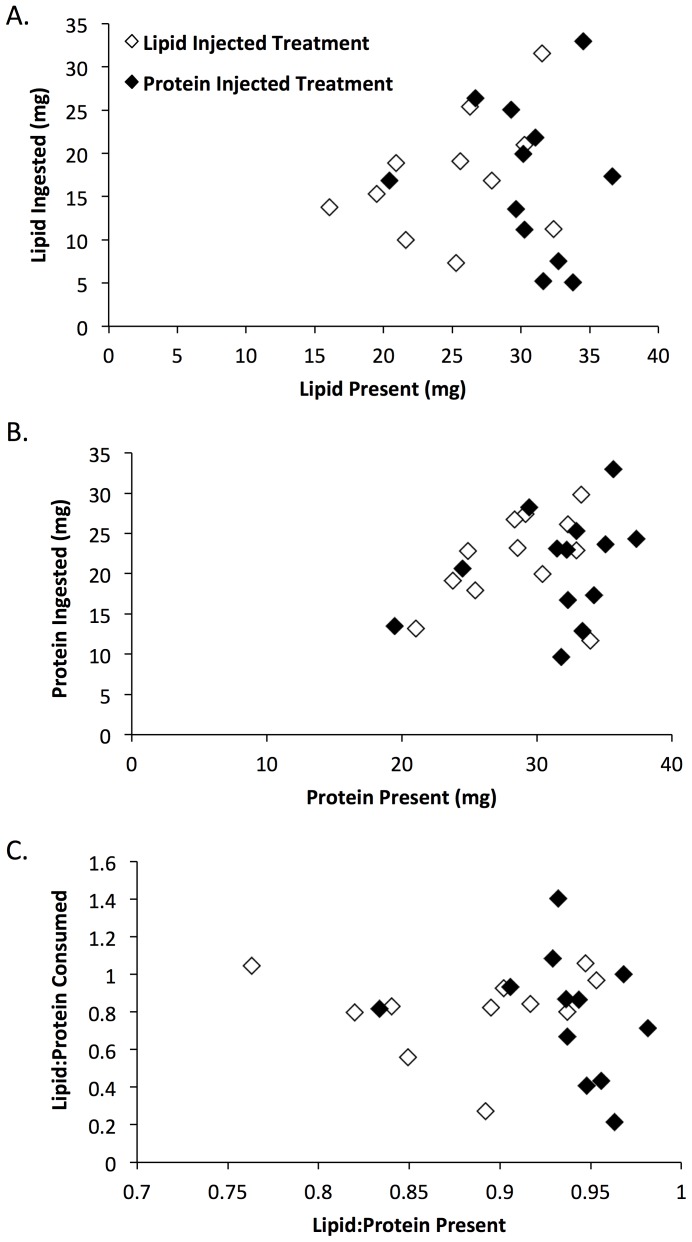
Nutrient extraction by spiders feeding on unmanipulated locust 2. For the second unmanipulated locust, tests for differential extraction of A) lipid, B) protein, and C) the ratio of lipid to protein from the locust body by *Argiope keyserlingi* spiders. All spiders had previously been fed two locusts that were injected with nutrient solutions and one unmanipulated locust. No regression lines are plotted because there were no significant linear relationships between independent and dependent variables.

For the second unmanipulated locust, there were no differences between treatments in the relationships between lipid present in a locust and lipid consumed by the spider (F_1,19_ = 0.1, p = 0.77), protein present in the locust and protein consumed by the spider (F_1,21_ = 0.8, p = 0.39) and the ratio of lipid to protein present in a locust and that consumed by the spider (F_1,19_ = 0.1, p = 0.87; Figure 4). For the second unmanipulated locust, there were no significant linear relationships between nutrients present and nutrients consumed by spiders (Figure 4; protein: F_1,21_ = 2.8, p = 0.11; lipid: F_1,19_ = 0.11, p = 0.74; ratio: F_1,19_ = 0.5, p 0.47). Spiders consumed an overall lower and more variable proportion of nutrients from these second unmanipulated locusts (Figure 4; 71+4% of available protein, and 63+6% of available lipid) compared to the first unmanipulated locusts.

## Discussion

The results support the hypothesis that web-building spiders, *A. keyserlingi*, do not demonstrate strict nutritional homeostasis but, rather, are flexible in nutrient intake and storage. The nutrient content of the bodies of spiders was directly related to the nutrient content of their prey. The lipid and protein content of the spider's bodies increased independently depending on the amount of each nutrient present in prey. In addition, spiders did not show any evidence of selective nutrient extraction from large prey when they had previously been fed nutritionally-biased prey (i.e., either high lipid or high protein). These results suggest that the web-building spider, *A. keyserlingi*, does not maintain nutritional homeostasis in body composition or nutrient intake, which is in contrast to the findings for many other animals [1], [2], [6].

Evidence suggests that some spiders are able to differentially extract particular nutrients from a whole prey item. Mayntz et al. [8] found that the spider *Stegodyphus lineatus* varied the amount of nitrogen relative to carbon extracted from a prey item depending on their prior diet. Individuals previously fed high lipid prey extracted relatively more nitrogen from prey relative to spiders that had been previously fed high protein prey; although, the effect size was relatively small [8]. Jensen et al. [12] also found evidence for differential lipid:protein extraction by wolf spiders feeding on the first fly in the most lipid-rich of six diet treatments. Yet in the current study, there was no evidence of selective nutrient extraction by *A. keyserlingi* when feeding on large prey after having been fed either high protein or high lipid prey. For the first large prey, the amount of lipid and protein eaten was linearly related to the amount of each nutrient present and not significantly different among treatments. For the second large prey, there were no linear relationships between nutrients present in prey and those consumed by spiders and also no differences between treatments. The lack of a linear relationship between nutrients present and those consumed for the second prey was likely because some spiders became satiated and ate less overall of the prey body. In addition, variation in the lipid:protein consumed by spiders (Figure 4C) was likely due to variation in the location of the locust body (e.g., abdomen  =  higher lipid, legs and thorax  =  higher protein) at which the spider began feeding before becoming satiated [9].

In nature, web-building spiders likely have few opportunities to exercise selective nutrient extraction because they may be overall food limited and may rarely encounter prey that are so large that they cannot be fully consumed [11], [19], [20]. For example, the feeding level of web-building spiders *Erigone atra* in the field was equivalent to over one week of starvation in the laboratory [20]. Food limitation may select for a high degree of flexibility in nutrient storage and less selectivity in nutrient ingestion and assimilation in some carnivores, especially sedentary predators such as web-building spiders [19]. In addition to *A. keyserlingi*, wolf spiders, jumping spiders and praying mantids also appear flexible in the nutrient composition of their bodies depending upon the ratios of nutrients in their diet [12], [21], [22]. Hence, while many herbivores maintain a high degree of nutritional homeostasis [6], [7], carnivores may be more likely to exhibit flexibility in their body composition.

There are various physiological mechanisms through which animals can allocate nutrients either to maintain nutritional homeostasis or to vary in their body composition. Lipid is an obvious nutrient to store because it has a high energy density and can sustain metabolism during periods of food limitation. Many animals have specific tissues for lipid storage (e.g., fat body in insects). Many animals can also store protein as storage hexamers in the haemolymph or fat body [23]. In our study, web-building spiders, *A. keyserlingi*, were able to independently assimilate both lipid and protein in their bodies. In addition to specific tissues for lipid and protein storage, these spiders may have varied the nutrient content of their bodies through allocation of nutrients to the ovaries or developing eggs. A study of praying mantids showed that the lipid:protein content of eggs varied according to the lipid:protein content of the mantid diet [22]. While eggs and ovaries are not typically thought of as tissues for nutrient storage females are able to resorb nutrients from eggs to fuel metabolism if they experience food limitation [24]. Further work is needed to examine how variation in nutrient intake affects the allocation of nutrients to different tissues.

Our results confirm that nutrient injections provide an experimental method of quantitatively manipulating the nutrient content of live arthropod prey for predators. Nutritionally manipulated locusts varied predictably in nutrient content depending upon the amount of nutrients injected into their bodies and spiders assimilated these nutrients in the same ratios that were present in the prey. Recent work suggests that the nutrient content of prey can have large effects on carnivore growth, reproduction and survival and that nutrient-based prey choice can affect the structure and function of food webs [13], [18]. Experimental studies using nutrient injections into live prey and quantitative frameworks for analyzing diets and their consequences for animals can significantly advance our understanding of the connections between the physiological requirements of animals, the behavior they use to meet these requirements and the ecological consequences of diet and diet regulation behaviors [2].
